# Account of Diseases in an Eastern District of London, from April 20, to May 20, 1803

**Published:** 1803-06-01

**Authors:** 


### *Account of Diseases in an Eastern District of London, from April 20, to May 20, 1803.*

| | |
|---|---|
| ACUTE DISEASES. | |
| Synocha Catarrhalis | 10 |
| Typhus | 4 |
| Scarlatina Anginosa | 6 |
| CHRONIC DISEASES. | |
| Dyspnœa | 6 |
| Tussis | 10 |
| Tussis & Dyspnœa | 15 |
| Hæmoptysis | 4 |
| Pleurodyne | 3 |
| Anasarca | 3 |
| Ophthalmia | 2 |
| Amenorrhœa | 7 |
| Chlorosis | 5 |
| Fluor Albus | 4 |
| Menorrhagia Difficilis | 3 |
| Dyspepsia | 6 |
| Hypochondriasis | 2 |
| Ecchymosis | 1 |
| Dysuria | 2 |
| Hæmorrhagia Intestinalis | 2 |
| Hæmorrhois | 4 |
| Hernia | 1 |
| Paralysis | 3 |
| Nephralgia | 2 |
| Sciatica | 2 |
| Rheumatismus Chronicus | 25 |
| PUERPERAL DISEASES. | |
| Peritonitis | 3 |
| Ephemera | 4 |
| Menorrhagia Lochialis | 7 |
| Mastodynia | 8 |
| INFANTILE DISEASES. | |
| Aphthæ | 5 |
| Ophthalmia | 3 |
| Anus Imperforatus | 1 |
| Herpes | 5 |
| Vermes | 4 |

The number of patients labouring under the late epidemic disease has been gradually diminishing, and at present the subjects of it are but very few. In many instances, however, the period of convalescence has been much protracted; and during the debility which has prevailed in consequence of it, patients have been liable to the attack of some chronic disease, which has proved more obstinate and tedious than usual.

This remark applies more particularly to Chronic Rheumatism, numerous cases of which have occurred, that have resisted the usual means of cure.

CRITICAL

